# The mediating role of basic psychological needs satisfaction in the link between parental expectations and competitive state anxiety among adolescent tennis players

**DOI:** 10.3389/fpsyg.2026.1757414

**Published:** 2026-05-07

**Authors:** Ziyang Zhang, Zhaoyuan Chen, Lu Peng

**Affiliations:** 1 Department of Physical Education, Foshan University, Foshan, Guangdong Province, China; 2 Kangwon National University, Chuncheon-si, Gangwon-do, Republic of Korea

**Keywords:** competence satisfaction, competitive state anxiety, mediation analysis youth sports, parental expectations, self-determination theory

## Abstract

**Introduction:**

Pre-competitive anxiety is a critical psychological factor affecting adolescent athletes’ performance and well-being. Although perceived parental expectations have been linked to athletes’ anxiety, the psychological mechanisms underlying this association remain insufficiently understood. Grounded in Self-Determination Theory and Multidimensional Anxiety Theory, this study examined whether basic psychological needs satisfaction mediates the relationship between perceived parental expectations and pre-competitive state anxiety among adolescent tennis players.

**Methods:**

A total of 492 junior high school tennis players (*M*_age_ = 13.2 years) completed the Parental Expectations Questionnaire, the Basic Psychological Needs Satisfaction Scale, and the Competitive State Anxiety Inventory-2. Structural equation modeling and the SPSS PROCESS macro were used to test the mediation model, with bias-corrected bootstrapping applied to estimate indirect effects.

**Results:**

Perceived parental expectations significantly and positively predicted pre-competitive state anxiety (*β =* 0.153, *p =* 0.001). Basic psychological needs satisfaction partially mediated this relationship, with parental expectations negatively predicting overall needs satisfaction (*β =* −0.147, *p =* 0.010). Parallel multiple mediation analysis further showed that, among the three basic psychological needs, only competence satisfaction served as a significant and stable indirect pathway (*β =* 0.014, 95% CI [0.001, 0.034]). Although perceived parental expectations also negatively affected autonomy satisfaction, the indirect effects through autonomy and relatedness were not statistically significant.

**Discussion:**

These findings suggest that elevated parental expectations may increase adolescent athletes’ pre-competitive anxiety primarily by undermining their perceived competence. The results highlight the importance of reducing rigid performance-oriented parental pressure and fostering a mastery-oriented coaching environment that supports athletes’ competence, self-efficacy, and psychological readiness before competition.

## 1 Introduction

Youth sport participation offers a multitude of physiological and psychosocial benefits, including enhanced cardiovascular health, character development, and the fostering of peer relationships (Côté and Hancock, 2016; Gould and Westfall, 2014). However, the highly evaluative nature of competitive athletics inherently exposes adolescent athletes to significant psychological stress. A primary manifestation of this stress is competitive state anxiety, defined as an acute, immediate emotional response to a specific competitive situation (Martens et al., 1990). According to Multidimensional Anxiety Theory, this state anxiety comprises cognitive worry, somatic physiological arousal, and a disruption of state self-confidence (Smith et al., 2006). When athletes are unable to effectively regulate these acute anxiety symptoms, they frequently experience performance decrements, diminished athletic enjoyment, and a heightened risk of eventual sport dropout (Crane and Temple, 2015; Gustafsson et al., 2017). Consequently, identifying the antecedents and underlying psychological mechanisms that exacerbate pre-competitive anxiety remains a critical priority within sport psychology research.

While competitive anxiety manifests internally, its developmental roots are deeply embedded in the athlete’s psychosocial environment. For adolescent athletes, the family unit serves as the most proximal and influential socializing agent (Fredricks and Eccles, 2004). Supportive parental involvement has been consistently linked to autonomous motivation and sustained athletic engagement (Knight et al., 2010; Sánchez-Miguel et al., 2013). Conversely, when parental involvement is characterized by pervasive, rigid, and outcome-oriented demands, it ceases to be a supportive mechanism and transforms into a potent environmental stressor (Harwood and Knight, 2015). In this context, it is critical to distinguish between normative expectations and the psychological pressure derived from excessive demands. Within the framework of Self-Determination Theory, rigid parental expectations function as a primary source of external pressure, especially when adolescent athletes perceive that parental affection and approval are contingent solely upon athletic success (Appleton et al., 2010; Assor et al., 2004). Adolescents subjected to these uncompromising standards frequently internalize an intense fear of failure and appraise impending competitions as severe psychosocial threats rather than manageable challenges (Lazarus, 2000). Although previous literature has established a broad link between parental pressure and general athlete distress (O'Rourke et al., 2014), the precise psychological conduits through which distal parental expectations translate into acute, pre-competitive state anxiety remain inadequately understood.

Self-Determination Theory (SDT) provides a robust theoretical framework to elucidate this complex internalization process (Ryan and Deci, 2017). SDT posits that human flourishing and optimal emotional regulation depend upon the satisfaction of three innate basic psychological needs: autonomy, competence, and relatedness (Deci and Ryan, 2000). The social environment plays a critical role in either nourishing or thwarting these needs. In the context of youth sports, controlling environments characterized by excessive external demands systematically deplete these psychological resources (Bartholomew et al., 2011). When parents impose unrealistic expectations, athletes experience a loss of volitional freedom (thwarted autonomy) and internalize a persistent sense of inadequacy (thwarted competence), effectively shifting their athletic motivation from intrinsic joy to external obligation (Vansteenkiste and Ryan, 2013).

According to the stress-buffering perspective of SDT, the fulfillment of basic psychological needs equips individuals with a cognitive shield capable of mitigating the effects of external stressors (Weinstein and Ryan, 2011). Therefore, when adolescent athletes perceive their needs as thwarted by demanding parents, they enter the competitive arena in a state of heightened psychological vulnerability. Stripped of their internal regulatory resources, these athletes become highly susceptible to the cognitive worry and somatic arousal that define state anxiety (Blanchard et al., 2009; Smith et al., 2007).

Crucially, while the three psychological needs are theorized to operate synergistically for general wellbeing, their respective impacts may diverge in highly specific, hyper-evaluative contexts such as the immediate build-up to a sporting event. Sport is fundamentally a domain of physical and tactical evaluation (Amorose and Anderson-Butcher, 2007). Consequently, an athlete’s perceived ability to execute skills under pressure may supersede broader relational or autonomous concerns in the moments preceding a match (Feltz and Magyar, 2006). If parental expectations primarily attack an athlete’s belief in their own capabilities, the thwarting of competence may emerge as the most critical and direct catalyst for pre-competitive cognitive distress. However, empirical studies examining the parallel mediating roles of all three distinct psychological needs between parental expectations and state anxiety are notably absent from the literature.

To address this gap, the present study aims to elucidate the structural mechanisms linking perceived parental expectations to competitive state anxiety in adolescent athletes. By integrating Multidimensional Anxiety Theory and Self-Determination Theory, we propose a parallel multiple mediation model to isolate the specific psychological pathways driving this relationship.

Based on the theoretical frameworks and empirical evidence reviewed above, the following hypotheses were formulated:

*Hypothesis 1 (H1):* Perceived parental expectations will exhibit a significant direct and positive effect on competitive state anxiety.

*Hypothesis 2 (H2):* Perceived parental expectations will significantly and negatively predict the satisfaction of basic psychological needs.

*Hypothesis 3 (H3):* Basic psychological needs satisfaction will significantly and negatively predict competitive state anxiety, acting as a protective psychological buffer.

*Hypothesis 4 (H4):* Basic psychological needs satisfaction will partially mediate the relationship between parental expectations and competitive state anxiety. Specifically, within a parallel mediation framework, the thwarted need for *competence* is hypothesized to emerge as the dominant and statistically robust mediating pathway compared to autonomy and relatedness, reflecting the hyper-evaluative nature of the pre-competitive sport context.

**Figure 1:**
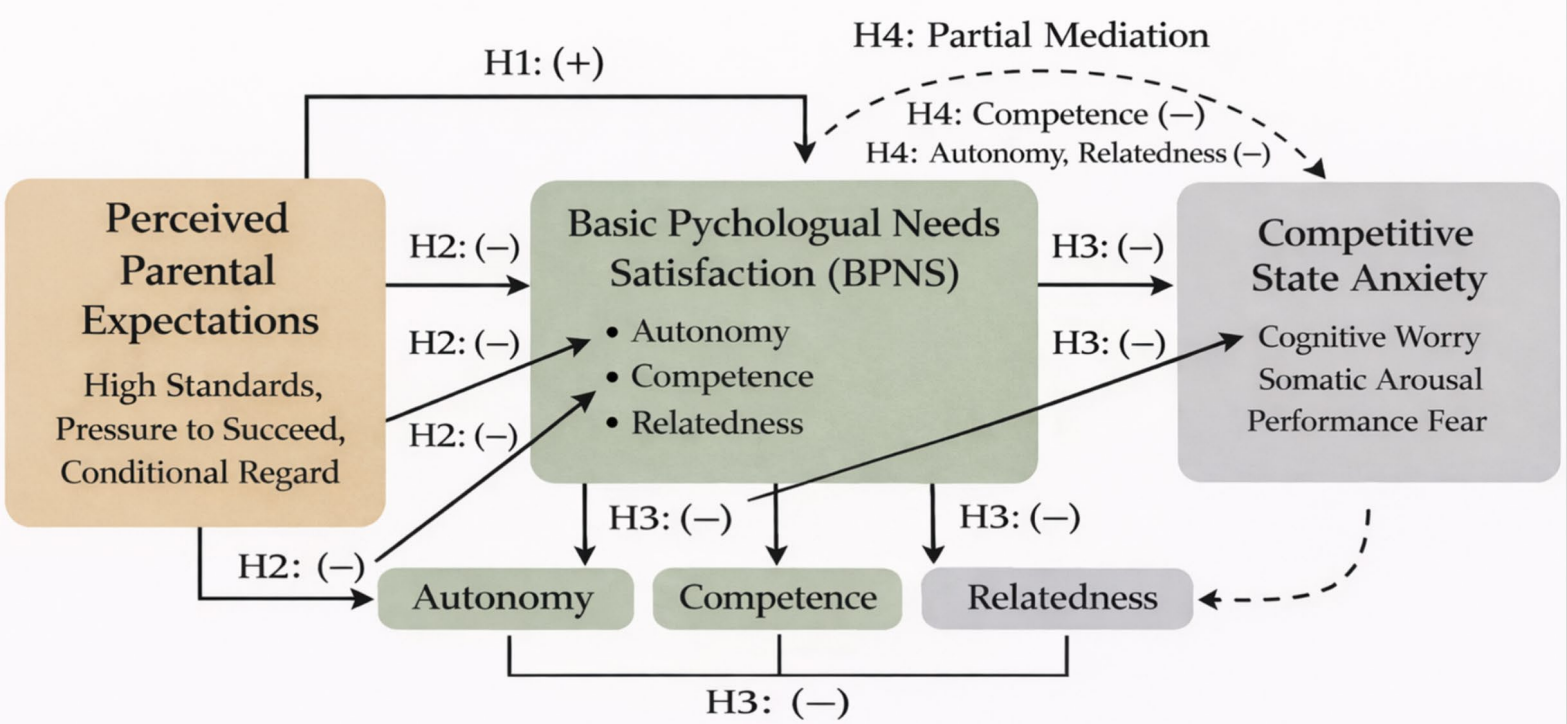
Research hypothesis model. The diagram illustrates the proposed structural relationships based on self-determination theory and multidimensional anxiety theory. Rectangles represent the core constructs measured in the study. Solid arrows indicate direct predictive pathways. Dashed arrows indicate the hypothesized indirect (mediation) effect (H4). (+) denotes a hypothesized positive correlation. (−) denotes a hypothesized negative correlation; higher needs satisfaction reduces anxiety. H1–H4 correspond to the specific research hypotheses detailed in the text.

Figure 1 illustrates the integrated conceptual framework of the present study. This diagram visually delineates the hypothesized structural pathways between perceived parental expectations, basic psychological needs satisfaction, and competitive state anxiety. As shown in the model, perceived parental expectations are positioned as the primary independent variable. Hypothesis 1 represents the direct positive relationship between these expectations and state anxiety.

Furthermore, the model highlights the mediating role of the three basic psychological needs. The pathways labeled H2 reflect the negative impact of parental pressure on the satisfaction of autonomy, competence, and relatedness. Correspondingly, the pathways labeled H3 indicate that the fulfillment of these needs is expected to act as a protective buffer that inversely predicts anxiety outcomes. Finally, the dashed line representing Hypothesis 4 illustrates the overall partial mediation mechanism, specifically highlighting the parallel indirect pathways through which parental expectations influence competitive state anxiety via the satisfaction of the three distinct psychological needs.

The overarching aim of this research is to clarify the predictive relationship between perceived parental expectations and competitive state anxiety among adolescent athletes. Specifically, this study seeks to determine the direct structural effect of controlling parental demands on cognitive and somatic anxiety while simultaneously evaluating the parallel mediating mechanisms provided by autonomy, competence, and relatedness satisfaction. By addressing these specific aims, we intend to provide a more granular understanding of how family-level stressors are internalized and manifested as psychological distress in competitive sport settings. This investigation ultimately serves to bridge the gap between distal parental influences and acute pre-competitive emotional responses.

## 2 Methods

### 2.1 Participants

Participants were recruited via cluster random sampling from junior high school tennis teams located in the Guangdong and Guangxi provinces of China. A total of 500 surveys were originally disseminated. Following a rigorous data screening process, responses exhibiting incomplete information or patterned answering were removed. This resulted in a final dataset of 492 valid responses, corresponding to an effective response rate of 98.4%.

The final analytical sample (*N* = 492) consisted of adolescent athletes actively serving on regional representative youth teams. This cohort included a diverse age range, encompassing participants under the age of 12 (22.0%, *n =* 108) alongside those in middle and late adolescence. For the purposes of this study, regular professional training is defined as participation in systematic, high-intensity athletic programs conducted at regional training bases with the primary goal of competing in provincial or national events. In terms of gender, the sample comprised 233 males (47.4%) and 259 females (52.6%). The remaining age distribution was as follows: 32.5% (*n =* 160) were between 12 and 13 years old, 23.8% (*n =* 117) were between 14 and 15 years old, and 21.7% (*n =* 107) were 15 years of age or older.

### 2.2 Measures

#### 2.2.1 Parental expectations

To ensure the transparency and structural clarity of the measurement instrument, Table 1 presents the classification of the Parental Expectations Questionnaire along with representative item examples for each dimension. The 24-item scale is organized into five distinct thematic domains: Academic Performance (4 items), Future Development (4 items), Conduct Performance (3 items), Interpersonal Relations (6 items), and Physical/Mental Quality (7 items). As demonstrated by the provided examples, the items specifically assess the intensity of expectations that adolescent athletes perceive from their parents, ranging from specific scholastic achievement and future career attainment to moral behavior and psychosocial wellbeing. This detailed breakdown provides a clear conceptual foundation for the instrument, which was specifically adapted for the Chinese cultural and athletic context.

**Table 1:** Classification and item examples.

Dimension	Items	Representative item examples
Academic performance	4	“My parents expect me to rank among the top in my class.”
Future development	4	“My parents expect me to attain high social status and achievement in the future.”
Conduct performance	3	“My parents expect me to be an upright person and follow social rules.”
Interpersonal relations	6	“My parents do not care whether I maintain a good relationship with my peers at school.”
Physical/mental quality	7	“My parents hope that I have appropriate methods for relieving stress.”

**Figure 2:**
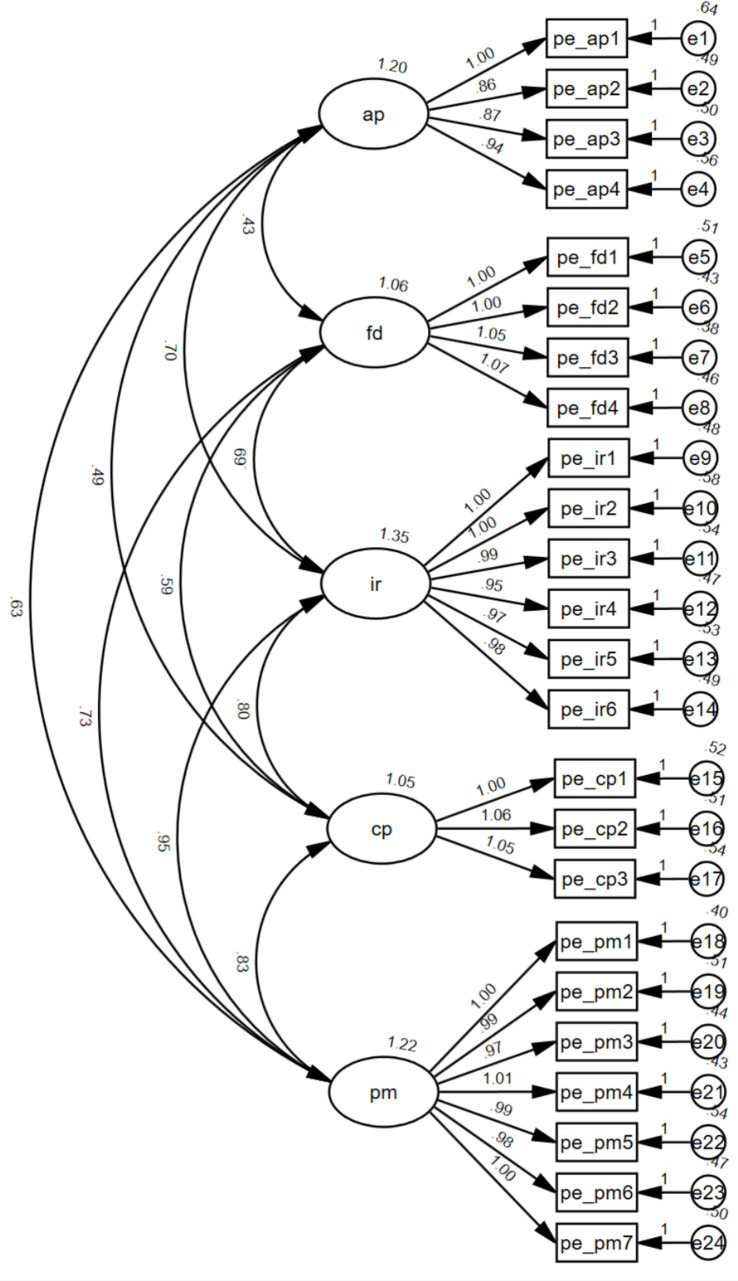
Measurement model of the parental expectations questionnaire.

In terms of reliability, the scale exhibited robust psychometric properties. Exploratory Factor Analysis (EFA) yielded a Kaiser-Meyer-Olkin (KMO) value of 0.954, suggesting excellent sampling adequacy. Internal consistency was exceptionally high for the total scale (Cronbach’s *α* = 0.957), with strong reliability across all sub-dimensions: Academic (*α* = 0.880), Future (*α* = 0.910), Interpersonal (*α* = 0.938), Conduct Performance (*α* = 0.866), and Physical/Mental (*α* = 0.947). Given that this instrument originates from a graduate thesis and lacks the extensive standardization of commercially established scales, a Confirmatory Factor Analysis (CFA) was deemed necessary to validate its structural integrity for the current study. Utilizing AMOS 26.0, we tested the measurement model (see Figure 2). The results demonstrated a favorable model fit, characterized by the following indices: $\chi^{2}$ = 318.32, df = 242, $\chi^{2}/\mathrm{df}$ = 1.32, CFI = 0.99, GFI = 0.95, RMR = 0.037, and RMSEA = 0.025. Furthermore, Pearson correlation analysis (Table 1) revealed that all five dimensions of the questionnaire were significantly and positively intercorrelated (*p <* 0.001), with coefficients (r) ranging from 0.337 to 0.700 (Table 2).

**Table 2:** Correlation analysis of the five dimensions of the parental expectations questionnaire.

Variables	ap	fd	cp	ir	pm
Academic Performance (ap)	1				
Future Development (fd)	0.337***	1			
Conduct Performance (cp)	0.381***	0.498***	1		
Interpersonal Relations (ir)	0.496***	0.535***	0.608***	1	
Physical/Mental Quality (pm)	0.477***	0.598***	0.666***	0.700***	1

***p < 0.001.

#### 2.2.2 Assessment of basic psychological needs satisfaction

The BPNS was selected because it is the most widely recognized instrument for assessing the universal psychological needs identified by Self-Determination Theory (Deci and Ryan, 2000). Its cross-cultural validity and applicability to youth sport settings have been extensively documented (e.g., Standage et al., 2005), making it a theoretically sound choice for capturing the psychological experiences of adolescent athletes in the Chinese context.

To gauge the degree to which athletes perceived their psychological requirements were being satisfied, the Chinese version of the Basic Psychological Needs Scale (Deci et al., 2001) was administered. This 19-item instrument encompasses three specific domains: Competence (6 items), Autonomy (5 items), and Relatedness (8 items). Responses were captured on a 7-point Likert scale (1 = completely non-conforming, 7 = very conforming). Psychometric evaluation in the current sample confirmed robust construct validity (KMO = 0.949). Internal consistency was high for the aggregate scale (*α* = 0.940) and consistent across sub-dimensions: Competence (*α* = 0.940), Autonomy (*α* = 0.924), and Relatedness (*α* = 0.960).

#### 2.2.3 Measurement of competitive state anxiety

The CSAI-2 was chosen as the “gold standard” for measuring state-specific competitive anxiety. Unlike general anxiety scales, the CSAI-2 was specifically developed for athletes to capture pre-performance distress (Martens et al., 1990). The localized Chinese version (Zhou, 2000a; Zhou, 2000b) has demonstrated high structural stability and sensitivity in junior cohorts, ensuring that the measurements are contextually relevant to the psychological states of young tennis players prior to competition. The inventory includes 27 items structured into three subscales: Cognitive State Anxiety (9 items), Somatic State Anxiety (9 items), and State Self-Confidence (9 items). Participants rated items on a 4-point intensity scale ranging from 1 (*not at all*) to 4 (*very much so*). Factor analysis suitability was confirmed with a KMO value of 0.933. Reliability was excellent for the overall measure (*α* = 0.953) and its components: Cognitive Anxiety (*α* = 0.927), Somatic Anxiety (*α* = 0.942), and Self-Confidence (*α* = 0.950).

### 2.3 Data collection and study protocol

Following ethical clearance from the institutional review board and relevant sports authorities, field research was initiated at the respective team training bases. To prioritize ecological validity and minimize retrospective bias, surveys were distributed strictly 1 h prior to scheduled competitions or simulated high-intensity matches. Recognizing the developmental diversity within the sample, trained research assistants provided age-appropriate instructions tailored to the cognitive stages of the participants.

Specifically, for the youngest cohort (aged under 12, *n* = 108), a guided completion protocol was implemented in small groups (3–5 individuals) to ensure focused attention. To mitigate potential variations in reading comprehension, research assistants read each item of the BPNS and CSAI-2 scales aloud. Assistants utilized simplified, standardized terminology to explain abstract psychological constructs. For instance, “autonomy” was illustrated as “feeling like you have a choice in your training,” and “cognitive anxiety” was described as “having worried thoughts about your performance before the match.” Before recording their responses, participants were asked to briefly paraphrase their understanding of selected items to confirm comprehension.

These younger participants were explicitly reassured that their individual responses would be kept strictly confidential and would not be disclosed to their parents or coaches. A dual-consent procedure was implemented throughout the study, whereby written informed consent was obtained from legal guardians while written or verbal assent was secured from the adolescent athletes themselves. During the administration process, research assistants remained available to clarify any linguistic or conceptual queries while strictly avoiding any influence on the participants’ independent judgment. Post-hoc reliability analyses confirmed that the internal consistency for the youngest subgroup remained robust (all sub-scale *α* coefficients > 0.80), ensuring the statistical integrity of the data across all age levels. The average completion time for the entire battery of questionnaires was approximately 15 min.

### 2.4 Statistical analytic strategy

Data management and analysis were executed using SPSS 27.0 and AMOS 27.0. The analytical framework consisted of three phases: (1) computation of descriptive statistics (means, standard deviations) to profile the sample; (2) verification of psychometric properties via reliability (Cronbach’s *α*) and validity tests (KMO, Bartlett’s Test); and (3) hypothesis testing using Structural Equation Modeling (SEM). The SEM analysis examined the structural relationships between parental expectations, needs satisfaction, and anxiety, with model fit assessed via standard indices (*χ*^2^/df, CFI, TLI, RMSEA). To ensure sufficient sensitivity for detecting mediating mechanisms, a power analysis was considered. Based on the criteria established by Fritz and MacKinnon (2007), our final sample of *N* = 492 provided adequate statistical power (exceeding 0.80) to detect small-to-medium indirect effects, ensuring that the non-significant findings for autonomy and relatedness were not primarily a result of insufficient sample size.

## 3 Results

### 3.1 Assessment of common method Bias

Given the cross-sectional design and reliance on self-reported measures for all constructs, the potential for common method variance (CMV) was acknowledged. To mitigate this risk, procedural controls—such as ensuring respondent anonymity and utilizing reverse-coded items—were integrated into the survey administration (Podsakoff et al., 2012). Furthermore, statistical verification was performed using Harman’s single-factor test. An unrotated exploratory factor analysis (EFA) including all measurement items was conducted. The analysis extracted multiple distinct factors with eigenvalues exceeding 1.0. Crucially, the first unrotated factor accounted for only 21.49% of the total variance. This value falls significantly below the widely accepted threshold of 50% (Tehseen et al., 2017), suggesting that common method bias is not a pervasive issue in this dataset and does not confound the subsequent interpretations.

### 3.2 Descriptive statistics and inter-correlations

Table 3 summarizes the descriptive statistics (Means, SD) and the Pearson correlation matrix for the three core variables: Parental Expectations, Basic Psychological Needs Satisfaction (BPNS), and Competitive State Anxiety. As hypothesized, the analysis revealed significant correlations among all key constructs. The direction and magnitude of these associations align with the theoretical framework, providing a preliminary basis for the structural equation modeling.

**Table 3:** Correlation analysis of variables between competitive state anxiety and parental expectations.

Variables	ap	fd	ir	cp	pm	nc	na	nb	csa	psa	sc
Academic Performance (ap)	–										
Future Development (fd)	0.337**	–									
Interpersonal Relations (ir)	0.496**	0.535***	–								
Conduct Performance (cp)	0.381***	0.498***	0.608***	–							
Physical/Mental Quality (pm)	0.477***	0.598***	0.700***	0.666***	–						
Competence Needs (nc)	−0.101*	−0.076	−0.059	−0.179***	−0.152***	–					
Autonomy Needs (na)	−0.008	−0.132**	−0.046	−0.104*	−0.103*	0.373***	–				
Relatedness Needs (nb)	−0.012	−0.022	−0.072	−0.079	−0.094*	0.408***	0.470***	–			
Cognitive State Anxiety (csa)	0.071	0.027	0.041	0.123**	0.064	−0.146**	−0.120**	−0.115*	–		
Somatic State Anxiety (psa)	0.115*	0.116*	0.121**	0.177***	0.125**	−0.122**	−0.116*	−0.083	0.376***	–	
State Self-Confidence (sc)	0.121**	0.145**	0.089*	0.118**	0.153***	−0.139**	−0.151***	−0.085	0.516***	0.578***	
M (Mean)	3.11	3.17	3.16	3.18	3.22	4.03	3.99	4.02	2.36	2.37	2.42
SD (Standard Deviation)	1.07	1.12	1.18	1.14	1.13	1.38	1.43	1.50	0.76	0.84	0.88

*p < 0.05, **p < 0.01, ***p < 0.001.

Significant positive intercorrelations were observed among all five dimensions of parental expectations (i.e., academic performance, future development, interpersonal relations, conduct performance, and physical/mental quality), with coefficients ranging from r = 0.337 to 0.700 (all *p <* 0.01). This robust internal consistency suggests a pervasive generalizing effect; parents who impose high standards in one specific domain (e.g., academics) are highly likely to extend these expectations to other areas, thereby cultivating a comprehensive system of external pressure.

In alignment with Self-Determination Theory (SDT), parental expectations were generally found to be negatively associated with basic psychological needs satisfaction. Specifically, expectations regarding physical/mental quality exhibited significant negative correlations with the need for competence (r = −0.152, *p <* 0.001), autonomy (r = −0.103, *p <* 0.05), and relatedness (r = −0.094, *p <* 0.05). Similarly, expectations concerning conduct were inversely related to both competence (r = −0.179, *p <* 0.001) and autonomy (r = −0.104, *p <* 0.05). These findings imply that excessive external demands may operate as a controlling environmental factor, potentially thwarting athletes’ perceptions of autonomy and competence (Bartholomew et al., 2010).

Furthermore, the three subscales of BPNS—competence, autonomy, and relatedness—demonstrated moderate-to-strong positive intercorrelations (r = 0.373 to 0.470, all *p <* 0.001). This pattern indicates that the satisfaction of basic psychological needs typically occurs synergistically; when athletes feel a sense of competence, they are simultaneously more likely to experience volitional freedom and social connectedness.

Regarding competitive state anxiety, needs satisfaction demonstrated clear protective associations. The need for competence was significantly and negatively correlated with both cognitive anxiety (r = −0.146, *p <* 0.01) and somatic anxiety (r = −0.122, *p <* 0.01). Interestingly, it also exhibited a negative correlation with the state self-confidence dimension (r = −0.139, *p <* 0.01). It is crucial to note that because the state self-confidence subscale was reverse-scored in the present study, higher scores on this dimension inherently reflect lower levels of actual self-confidence. Additionally, cognitive and somatic anxiety were moderately positively correlated (r = 0.376, *p <* 0.001), and both were positively associated with the reverse-scored self-confidence dimension (r = 0.516 and 0.578, respectively; *p <* 0.001). Broadly, these empirical findings corroborate Multidimensional Anxiety Theory (Martens et al., 1990), confirming that cognitive distress and physiological arousal are distinct yet inextricably linked components of the pre-competitive experience.

**Figure 3:**
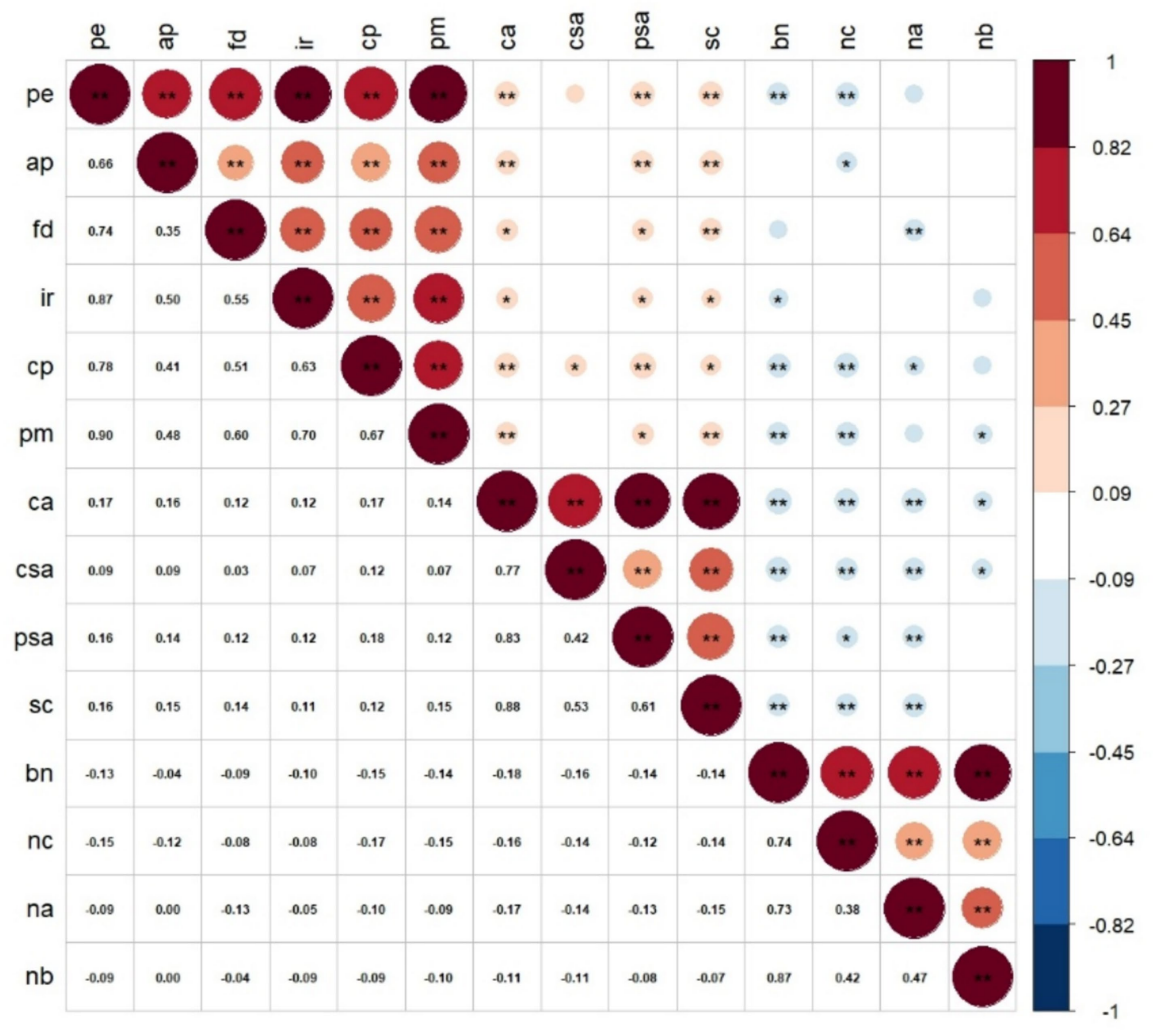
Heatmap matrix of bivariate associations across core study dimensions. This correlogram visualizes the directionality and magnitude of Pearson’s correlation coefficients (r). The chromatic scale transitions from dark blue (denoting pronounced negative correlations) to dark red (denoting pronounced positive correlations). Both color saturation and node diameter are scaled proportionally to the absolute effect size of the relationships. PE, Parental Expectations (ap, Academic Performance; fd, Future Development; ir, Interpersonal Relations; cp, Conduct Performance; pm, Physical/Mental Quality); BPNS, Basic Psychological Needs Satisfaction (nc, Competence; na, Autonomy, nb, Relatedness); CSAI-2, Competitive State Anxiety (csa, Cognitive Anxiety; psa, Somatic Anxiety; sc, State Self-Confidence).

To visually explore the complex interrelationships and evaluate the practical significance of the effect sizes, a correlogram was generated (Friendly, 2002), with association magnitudes interpreted based on contemporary psychological guidelines (Funder and Ozer, 2019). A diagrammatic summary of the bivariate correlations across all assessed variables is presented in Figure 3. An examination of the correlation matrix highlights concentrated clusters of strong intra-scale associations along the diagonal. This high degree of internal convergence—most notably among the sub-components of both Parental Expectations and Needs Satisfaction—further affirms the structural validity of the selected instruments. More importantly, the off-diagonal sectors visually capture the anticipated theoretical pathways. The warm-colored matrices (red hues) denote significant positive linkages between perceived parental pressure and state anxiety indicators. In stark contrast, the transition to cooler regions (blue hues) underscores the buffering role of basic psychological need fulfillment, evidenced by its distinct inverse correlations with both external parental demands and pre-competitive anxiety.

### 3.3 Analysis examination of structural pathways and mediating mechanisms

To unpack the underlying psychological mechanisms linking parental pressure to pre-performance distress, we conducted a mediation analysis using the SPSS PROCESS macro (Model 4; Hayes, 2022). While Figure 3 illustrates the broader structural relationships estimated via Maximum Likelihood, this specific analysis formally tested the mediating role of Basic Psychological Needs Satisfaction (BPNS) between parental expectations and cognitive anxiety, whilst adjusting for demographic covariates (Cheng, 2010). Acknowledging the substantial neurobiological maturation and cognitive shifts that occur across early and middle adolescence (Shaw et al., 2006), we strictly included age, gender, and training years as covariates in all mediation models. This procedure ensures that the observed relationships between parental pressure and anxiety are not confounded by the developmental heterogeneity of our sample.

Initial regression analyses confirmed that, in the absence of the mediator, parental expectations positively and significantly predicted cognitive anxiety (Total Effect Model: *R*^2^ = 0.029, *F*(5,486) = 2.90, *p =* 0.014; *β =* 0.113, t = 3.37, *p <* 0.001, 95% CI [0.047, 0.178]). Furthermore, parental expectations emerged as a significant negative predictor of BPNS fulfillment (*β = −*0.147, t = *−*2.59, *p =* 0.010, 95% CI [*−*0.259, *−*0.035]; model *R*^2^ = 0.019, *F*(5,486) = 1.87, *p =* 0.098).

**Figure 4:**
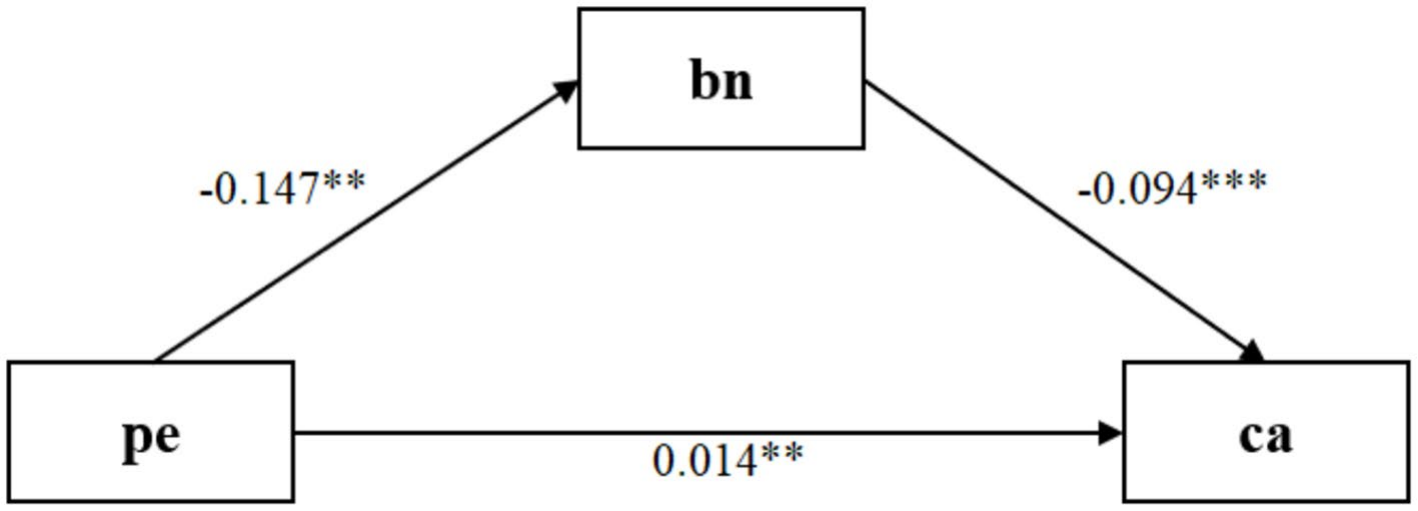
Standardized structural pathways linking parental expectations, needs satisfaction, and state anxiety. The depicted model adjusts for covariates including gender, age, and years of training experience (omitted from the diagram for visual simplicity). Ellipses denote latent constructs, whereas rectangles indicate observed indicators. Path estimates are presented as standardized coefficients. PE, Parental Expectations; bn, Basic Psychological Needs Satisfaction; ca, Competitive State Anxiety. Solid directional arrows represent significant predictive pathways (**p < 0.01, ***p < 0.001).

Upon introducing BPNS into the regression framework, the model’s explanatory power significantly improved [R^2^ = 0.054, *F*(6,485) = 4.61, *p <* 0.001]. Within this full model, BPNS negatively predicted cognitive anxiety (*β* = *−*0.094, t = *−*3.57, *p <* 0.001, 95% CI [*−*0.146, *−*0.042]). Concurrently, the direct effect of parental expectations on cognitive anxiety remained statistically significant, albeit slightly reduced (*β =* 0.099, t = 2.97, *p =* 0.003, 95% CI [0.033, 0.164]) (Figure 4).

To rigorously evaluate the significance of the mediation pathway, a bias-corrected bootstrapping procedure utilizing 5,000 resamples was executed (Preacher and Hayes, 2008). The analysis yielded a significant indirect effect of parental expectations on cognitive anxiety via BPNS ( β = 0.014, BootSE = 0.008). Crucially, the 95% confidence interval [0.002, 0.031] did not contain zero. Collectively, these findings establish a partial mediation mechanism: elevated parental expectations not only directly exacerbate cognitive anxiety but also indirectly amplify this distress by thwarting the satisfaction of the athletes’ basic psychological needs.

To further delineate the specific structural mechanisms at play, a parallel multiple mediation analysis was executed using the SPSS PROCESS macro (Model 4; Hayes, 2022). This procedure allowed for the simultaneous examination of the three distinct need satisfaction dimensions—competence (nc), autonomy (na), and relatedness (nb)—as potential mediators. In accordance with the developmental considerations previously noted (Shaw et al., 2006), all estimated paths adjusted for age, gender, and training experience as covariates. Table 4 summarizes the decomposition of the total, direct, and specific indirect effects derived from a bias-corrected bootstrapping approach utilizing 5,000 resamples.

**Table 4:** Decomposition of effects and parallel mediation pathways from parental expectations to competitive state anxiety.

Effects from PE to CA	Total effect (95% CI)	Direct effect (95% CI)	Total indirect effect (95% CI)
Standardized β	0.153*** (0.047, 0.178)	0.128** (0.029, 0.160)	0.025* (0.005, 0.050)
Specific Mediating Pathways (M)	PE → M ( β )	M → CA ( β )	Specific Indirect Effect ( β , 95% CI)
Competence (nc)	−0.136**	−0.104*	0.014 (0.001, 0.034)*
Autonomy (na)	−0.096*	−0.102*	0.010 (−0.001, 0.028)
Relatedness (nb)	−0.062	−0.010	0.001 (−0.007, 0.010)

PE, Parental Expectations; CA, Competitive State Anxiety. Estimates are standardized path coefficients ( β ). The 95% confidence intervals (CI) for indirect effects were generated using bias-corrected bootstrapping with 5,000 resamples. A specific indirect effect is considered significant if its CI does not straddle zero (highlighted in bold). *p < 0.05, **p < 0.01, ***p < 0.001.

Initial regression analyses confirmed that, in the absence of the mediator, controlling parental expectations positively predicted cognitive anxiety (Total Effect Model: R = 0.029, *F*(5,486) = 2.90, *p* = 0.014; *β* = 0.113, t = 3.37, *p* < 0.001, 95% CI [0.047, 0.178]). Furthermore, parental expectations emerged as a significant negative predictor of overall BPNS fulfillment (*β* = −0.147, t = −2.59, *p* = 0.010, 95% CI [−0.259, −0.035]; model *R*^2^ = 0.019). Upon introducing the parallel mediators into the regression framework, the model’s explanatory power for cognitive anxiety significantly improved (*R*^2^ = 0.054, *F*(8,483) = 3.44, *p* < 0.001). This addition resulted in a statistically significant increase in the variance explained (Δ*R*^2^ = 0.025, *p* < 0.001). Within this full model, the direct effect of parental expectations on anxiety remained significant (*β* = 0.128, *p* = 0.005, 95% CI [0.029, 0.160]).

Examination of the specific structural pathways demonstrated that parental expectations significantly and negatively predicted the satisfaction of competence (*β* = −0.136, *p* = 0.0003, 95% CI [−0.225, −0.047]) and autonomy (*β* = −0.096, *p* = 0.036, 95% CI [−0.224, −0.008]). When controlling for expectations and all covariates, both competence (*β* = −0.104, *p* = 0.039, 95% CI [−0.186, −0.005]) and autonomy (*β* = −0.102, *p* = 0.047, 95% CI [−0.203, −0.001]) negatively predicted state anxiety.

The evaluation of the specific indirect effects via 5,000 bootstrapping resamples confirmed a significant total indirect effect (*β* = 0.025, 95% CI [0.005, 0.050]). Crucially, when partitioning this effect, only the sequence operating through competence was statistically robust (*β* = 0.014, BootSE = 0.008, 95% CI [0.001, 0.034]). The indirect trajectories via autonomy (95% CI [−0.001, 0.028]) and relatedness (95% CI [−0.007, 0.010]) were non-significant as their respective confidence intervals encompassed zero. In summary, among the three basic psychological needs, the thwarted need for competence functions as the primary stable partial mediator linking controlling parental pressure to pre-competitive state anxiety.

## 4 Discussion

The primary objective of the present study was to elucidate the underlying psychological mechanisms through which perceived parental expectations influence competitive state anxiety in adolescent athletes. Grounded in the complementary frameworks of Self-Determination Theory and Multidimensional Anxiety Theory, our empirical findings corroborate the hypothesized structural model. Specifically, the results demonstrate that elevated parental expectations act as a potent environmental stressor that directly exacerbates pre-competitive cognitive and somatic anxiety. Furthermore, this study identified the thwarting of basic psychological needs as a crucial mediating mechanism. A granular examination of the data revealed a highly specific structural pathway, indicating that the thwarted need for competence serves as the primary psychological conduit linking external parental pressure to internal state anxiety. Collectively, these findings provide novel insights into how family-level demands are internalized by young athletes, ultimately depleting the psychological resources necessary for optimal emotional regulation in high-stakes athletic contexts.

### 4.1 The direct amplification of competitive anxiety by parental expectations

The present study confirmed that elevated parental expectations act as a significant direct predictor of both cognitive and somatic competitive state anxiety in adolescent athletes. This finding corroborates our initial hypothesis and provides robust empirical support for Multidimensional Anxiety Theory (Martens et al., 1990). While the foundational frameworks of Multidimensional Anxiety Theory were extensively validated in adult athletic populations, the current results suggest a significant developmental analogy in younger cohorts. However, it must be acknowledged that the transition of these mechanisms from adulthood to adolescence requires further direct empirical verification to account for distinct maturational differences in emotional regulation. Within the framework of this research, perceived parental expectations are conceptualized specifically as a controlling variable rather than a normative evaluative standard (Soenens and Vansteenkiste, 2010). Unlike purely evaluative benchmarks that may offer constructive feedback, these controlling expectations involve psychological pressure and the imposition of rigid benchmarks that demand strict athlete compliance. The significant direct effect identified in our model suggests that such extreme parental pressure possesses an independent and inherently toxic quality that can directly trigger physiological arousal and cognitive apprehension, even when bypassing the mediator of psychological needs (Hair et al., 2019).

The rationale for defining this construct as controlling is twofold. First, the content of the Parental Expectations Questionnaire specifically targets extrinsic markers of success, such as attaining high social status, earning significant wealth, and achieving superior class rankings. According to Self-Determination Theory (SDT), expectations centered on such external social comparisons are fundamentally aligned with a controlling interpersonal style, as they pressure the individual to meet externally imposed standards of worth (Assor et al., 2004). Second, the empirical evidence from our structural model further substantiates this controlling nature. The observation that parental expectations were significantly and negatively correlated with autonomy and competence satisfaction provides the definitive hallmark of a controlling climate. If the measured expectations were representative of autonomy-supportive involvement or healthy aspirations, they would theoretically be positively associated with need satisfaction rather than predicting need frustration (Hu and Bentler, 1999).

While the family unit serves as the primary socialization environment, it is critical to distinguish between different styles of parental involvement to avoid overgeneralizing these findings. High levels of parental involvement, when provided in an autonomy-supportive manner that emphasizes effort and personal mastery, are widely recognized as catalysts for athletic enjoyment (Sánchez-Miguel et al., 2013). However, our results highlight the detrimental consequences when involvement operates through a controlling interpersonal style. When parental involvement is characterized by the rigid and pervasive expectations identified in our instrument, it functions as a potent environmental stressor rather than a supportive resource (Liu and Liang, 2009).

This transition from support to distress can be understood through the lens of cognitive appraisal. Adolescents who perceive uncompromising parental standards often internalize these demands as a comprehensive system of external pressure. Consequently, as a high-stakes competition approaches, these athletes are significantly more likely to appraise the evaluative nature of the event as a severe threat rather than a manageable challenge (Lazarus, 2000). Driven by an intense fear of failure and the anticipatory dread of disappointing their family members, athletes experience a profound cognitive burden. This cognitive worry is frequently accompanied by physiological tension, reflecting the somatic component of their psychological distress (Gustafsson et al., 2017; Smith et al., 2007).

### 4.2 The thwarting of basic psychological needs via controlling environments

In strict alignment with Self-Determination Theory (SDT; Ryan and Deci, 2017), our results established that perceived parental expectations act as a controlling environmental factor that significantly undermines the satisfaction of basic psychological needs. According to the foundational premise of SDT, the social context is instrumental in either nourishing or thwarting an individual’s innate psychological development. When adolescents are subjected to pervasive and rigid parental standards, the athletic environment transforms from a nurturing context of self-discovery into a highly evaluative and restrictive domain. Our empirical data revealed that such externally imposed pressures specifically deplete the adolescent athletes’ fundamental sense of autonomy and competence, leaving them psychologically vulnerable.

The systematic deprivation of autonomy occurs because athletes operating under heavy parental expectations experience a profound shift in their perceived locus of causality. Rather than participating in sports for intrinsic enjoyment or personal mastery, their engagement becomes externally regulated. This motivation is often driven by introjected pressures, such as avoiding guilt, preventing parental disappointment, or appeasing authority figures (Deci and Ryan, 2000). Consequently, the athletes’ volitional freedom is severely compromised. Concurrently, the need for competence is directly thwarted by the imposition of absolute and often unrealistic performance benchmarks. When athletes consistently fall short of these elevated parental standards, they internalize a persistent sense of inadequacy and self-doubt, which effectively erodes their foundational belief in their own athletic capabilities (Vansteenkiste and Ryan, 2013).

Interestingly, the structural model indicated that parental pressure did not significantly erode the need for relatedness. This nuanced finding points to a complex interpersonal dynamic within the family unit. Adolescent athletes may still perceive a structural level of familial connection and instrumental support, as parents who harbor high expectations are often deeply invested in their children’s athletic endeavors through time commitment and financial provision (Knight et al., 2010). However, the qualitative nature of this bond is highly conditional. Parental affection and approval are frequently perceived by the athlete to be contingent upon performance outcomes and strict adherence to parental standards, a phenomenon identified in developmental psychology as conditional regard (Assor et al., 2004). This dictates that while the physical presence of the parents remains intact, the emotional environment operates through psychological control, subtly coercing the athlete and frustrating their holistic psychological maturation (Bartholomew et al., 2011; Soenens and Vansteenkiste, 2010).

### 4.3 The bridging mechanism: needs satisfaction as a partial mediator

The current findings provide empirical support for a structural configuration in which basic psychological needs satisfaction functions as a significant bridge between controlling parental expectations and cognitive state anxiety. While the cross-sectional nature of this study precludes definitive causal conclusions, the results demonstrate that parental demands do not operate in a vacuum to produce competitive distress. Instead, these external pressures align with a theoretical framework of indirect influence by systematically draining the athlete’s internal psychological resources. According to the foundational principles of Self-Determination Theory (SDT), the fulfillment of basic psychological needs is essential for optimal emotional regulation and the maintenance of psychological less than under stress (Ryan and Deci, 2017).

This relationship can be further understood through the lens of psychological buffering. Theoretically, when adolescent athletes perceive that their basic needs are adequately satisfied, they possess a robust psychological reservoir that may mitigate the anxiogenic effects of performance evaluations. Conversely, when controlling parental standards are perceived to thwart these needs, athletes may experience a depletion of internal resources, leaving them vulnerable to cognitive worry and unable to effectively manage the anticipatory stress preceding a match (Weinstein and Ryan, 2011). Although this buffering role of need satisfaction is well-documented in adult research, its application to the adolescent context remains an extrapolation that, while supported by our data, warrants caution. Adolescents possess evolving cognitive capacities that may alter how internal resources are deployed against external pressure compared to their adult counterparts. However, it is important to acknowledge the possibility of a reciprocal relationship. Athletes with higher baseline levels of anxiety may be cognitively predisposed to perceiving their social environment as more controlling, suggesting that the identified associations could be bidirectional in nature.

Crucially, the statistical identification of a partial mediation indicates that while the deprivation of psychological needs constitutes a significant pathway, the direct structural effect of parental expectations on anxiety remains robustly significant. This suggests that extreme parental pressure may possess an independent and inherently toxic quality that directly triggers physiological arousal and cognitive apprehension, bypassing the psychological needs framework altogether (Smith et al., 2006). The presence of these dual pathways confirms that parental expectations operate simultaneously as a direct environmental stressor and an indirect resource-depleting mechanism. While our model follows the established theoretical sequence of Self-Determination Theory, these results should be viewed as one plausible configuration among several competing structural alternatives, requiring future longitudinal validation to confirm the precise directional flow of these psychological processes (Scherer et al., 2017).

### 4.4 The distinctive dominance of competence in the evaluative sport context

Perhaps the most compelling and nuanced finding emerged from the parallel multiple mediation analysis. Among the three fundamental psychological needs, only the need for competence served as a statistically robust mediator bridging parental expectations and state anxiety. Although the structural model confirmed that parental expectations also thwarted the athletes’ sense of autonomy, the specific indirect pathway via autonomy to pre-competitive anxiety failed to reach statistical significance. Crucially, as established in our power analysis based on the criteria of Fritz and MacKinnon (2007), our sample possessed sufficient sensitivity to detect even small-to-medium indirect effects. Therefore, the non-significance of the autonomy and relatedness pathways is likely representative of their less proximal influence in this specific context rather than a lack of statistical power.

The prominent mediating role of competence should also be interpreted within the cultural and developmental context of the sample. While Self-Determination Theory maintains the universality of all three basic psychological needs (Ryan and Deci, 2017), the specific symptomatic manifestation of their frustration may be influenced by the collectivist orientation of Chinese society. In such cultures, individual success is frequently viewed through the prism of filial piety and familial obligation, where athletic achievement is inextricably linked to the preservation of family honor (Chen et al., 2015). Under the weight of controlling parental expectations, the athlete’s sense of competence becomes the primary psychological battlefield. The fear of being incapable of fulfilling these social and familial mandates triggers a more immediate cognitive anxiety than the restriction of personal choice. Furthermore, as adolescent athletes mature neurobiologically and cognitively, they acquire a greater capacity for abstract social comparison and a heightened sensitivity to evaluative pressure (Shaw et al., 2006). This suggests that the developmental progression from early to middle adolescence may amplify the perceived intensity of parental control due to the evolving cognitive appraisal of the familial implications of performance.

This distinction profoundly advances the application of SDT within sport psychology literature. It suggests a temporal and outcome-specific divergence in how thwarted needs manifest symptomatically. The frustration of competence directly attacks the athlete’s performance-related self-concept, acting as the immediate precursor to competitive cognitive anxiety (Blanchard et al., 2009). Consequently, athletes who internalize the belief that they lack the necessary skills to meet their parents’ stringent benchmarks are the most likely to experience debilitating cognitive distress when facing an imminent athletic evaluation. These results underscore that in achievement-oriented contexts, the erosion of perceived competence is the most critical psychological conduit linking distal parental pressure to acute pre-competitive distress.

Furthermore, it is important to consider the potential for perceptual bias among adolescent athletes. As suggested by the cognitive appraisal framework, individuals with lower baseline levels of perceived competence may be more predisposed to interpreting neutral or even well-intentioned parental aspirations as controlling pressure (Lazarus, 2000). This implies that the perceived ‘toxicity’ of parental expectations may be intensified by the athlete’s own psychological vulnerability, creating a reciprocal cycle of diminished competence and escalating anxiety.

### 4.5 Practical implications for parents and coaches

From an applied perspective, the findings of this study provide critical directives for optimizing the youth sport ecosystem. The practical significance of these results is underscored by the observation that psychological need satisfaction accounts for approximately 16.3% of the total impact that controlling parental expectations exert on competitive anxiety. While the absolute coefficient of the indirect effect may appear modest in a statistical sense, contemporary guidelines for effect size interpretation in the social sciences suggest that such associations are practically meaningful when they contribute to cumulative developmental outcomes (Funder and Ozer, 2019). Within the complex psychological landscape of adolescent athletics, a mechanism that explains over 16% of the variance in anxiety provides a substantial and targeted pathway for intervention.

For parents, these results highlight the urgent necessity of shifting the paradigm of involvement from expectation-driven pressure to autonomy- and competence-supportive parenting. Educational interventions should emphasize the detrimental psychological costs of imposing pervasive and rigid performance standards. Instead of focusing on competitive outcomes, parents must be guided to provide unconditional positive regard and to orient their feedback toward controllable metrics such as personal effort, resilience, and sportsmanship (Harwood and Knight, 2015). By moderating external demands and fostering an environment characterized by volitional freedom, parents can effectively reduce the primary external triggers of pre-competitive distress.

Furthermore, given that the thwarting of competence acts as the critical linchpin connecting external familial pressure to internal athlete anxiety, coaches possess a vital compensatory role. Our data identify competence satisfaction as a primary lever for strategic intervention. If an athlete arrives at a competition with depleted psychological resources due to demanding parents, the coach has the opportunity to actively restore the athlete’s sense of capability by cultivating a mastery-oriented motivational climate (Smith et al., 2007). By framing mistakes as essential learning opportunities and establishing individualized, achievable short-term goals, coaches can rebuild the athlete’s self-efficacy and buffer the anxiogenic effects of home-based stress.

Ultimately, mitigating competitive state anxiety requires a holistic and systemic approach to long-term athlete development. Sporting organizations and governing bodies must prioritize psychoeducational programs that target both the familial and coaching microsystems (Côté and Hancock, 2016). By fostering an environment where young athletes feel unconditionally valued and genuinely competent, stakeholders can safeguard adolescent mental health and promote lifelong, intrinsically motivated engagement in sports.

## 5 Limitations, future research, and practical implications

### 5.1 Limitations and future research directions

Despite the theoretical and empirical contributions of this study, several limitations must be acknowledged. A primary constraint involves the cross-sectional nature of the research design, which precludes the establishment of definitive causal relationships among parental expectations, needs satisfaction, and competitive state anxiety. Although the structural equation model was grounded in robust psychological theory, the observed associations remain correlational in nature. Consequently, future research should prioritize longitudinal designs or cross-lagged panel analyses to more accurately capture the temporal dynamics and directional influences of these psychological processes over a competitive season.

Furthermore, it is essential to specify the explanatory power of our current model to avoid overgeneralizing the results. Although the identified structural pathways are statistically significant, the variance explained in cognitive anxiety by parental expectations and psychological needs remains relatively modest at approximately 5.4%. This indicates that competitive distress in adolescent athletes is a multifaceted phenomenon influenced by a complex array of factors beyond the immediate family environment, such as coach-athlete relationships, peer dynamics, and individual personality traits. Subsequent studies should aim to integrate these diverse ecological levels to provide a more comprehensive account of the etiology of athletic anxiety.

Additionally, while psychometric evaluations supported the five-factor structure of the Parental Expectations Questionnaire, the high inter-correlation observed between certain dimensions—particularly Physical/Mental Quality and Interpersonal Relations (r = 0.70)—warrants a cautious interpretation. Although collinearity diagnostics (all VIFs < 2.7) confirmed that this overlap did not statistically bias the current mediation models, the strong association suggests that these domains may be perceived by adolescent athletes as a relatively unified cluster of external pressure. Future research should exercise caution when evaluating the independent effects of these highly related constructs and might consider exploring higher-order factor structures to further refine the conceptual boundaries of parental expectations in sports settings.

Another limitation pertains to the reliance on self-reported questionnaires from a single informant. While statistical tests indicated that common method variance was not a pervasive issue in this dataset, the use of athlete-only reports may still introduce social desirability bias. Subsequent investigations should consider a multi-informant approach by incorporating parental perspectives and coach evaluations to provide a more comprehensive and dyadic understanding of the family-sport interface. Furthermore, the current sample was localized to junior tennis players within a specific regional context. While this focus provided a controlled environment, it may limit the generalizability of the findings to team-based sports or different age cohorts. Replicating the model across a more diverse range of athletic disciplines and professional levels would be a valuable extension of the present work. The current study did not statistically test competing models such as reverse mediation. It is theoretically possible that a bidirectional relationship exists where high anxiety levels contribute to the frustration of psychological needs or heighten the sensitivity to parental demands. Without longitudinal data to establish temporal precedence, the identified dual-pathway should be viewed as one possible theoretical configuration among several competing structural alternatives. Future research utilizing cross-lagged panel designs would be invaluable in disentangling these complex, and potentially reciprocal, directional effects. A further conceptual limitation is that several theoretical parallels drawn in this discussion are based on empirical findings from adult samples. While our study observes a clear structural analogy between adult and adolescent psychological processes, we recognize that adolescents are in a unique stage of neurobiological and social development. Therefore, the findings reported here should be viewed as an important step toward adolescent-specific validation, and future research is needed to empirically confirm whether these adult-based models fully capture the developmental nuances of the youth sport experience.

### 5.2 Practical implications

The empirical evidence generated in this study offers several critical directives for practitioners and stakeholders involved in youth development. From a parental perspective, the results underscore the vital importance of transitioning from outcome-oriented expectations toward a parenting style that emphasizes autonomy and competence support. Parents should be encouraged to offer unconditional positive regard and to focus their feedback on controllable aspects of performance, such as effort and resilience, rather than absolute results or peer comparisons (Harwood and Knight, 2015). By moderating rigid standards and fostering an environment of volitional freedom, parents can reduce the external pressure that triggers pre-competitive distress.

Since the thwarting of competence was identified as the primary mediator linking external pressure to state anxiety, coaches possess a unique opportunity to act as a psychological buffer. When athletes perceive their competence is under threat from high parental demands, coaches can intervene by fostering a mastery-oriented motivational climate (Smith et al., 2007). This involves prioritizing individualized progress, viewing mistakes as essential learning opportunities, and establishing achievable short-term goals that reinforce the athlete’s sense of efficacy. Finally, sporting organizations should implement systemic psychoeducational programs targeting both parents and coaches. These initiatives should aim to cultivate a holistic sport ecosystem that prioritizes adolescent mental health and promotes long-term, intrinsically motivated engagement in competitive athletics (Côté and Hancock, 2016).

## 6 Conclusion

In conclusion, the present investigation provides a targeted empirical examination of the structural mechanisms through which controlling parental expectations precipitate competitive state anxiety among adolescent tennis players. Our findings confirm that while extreme parental pressure exerts a robust and direct influence on cognitive and somatic anxiety, this impact is further amplified through the systematic thwarting of basic psychological needs. Notably, the parallel mediation analysis identifies the satisfaction of competence as the primary psychological conduit within this relationship, highlighting the specific vulnerability of an athlete’s self-efficacy when subjected to rigid external benchmarks. By integrating Self-Determination Theory and Multidimensional Anxiety Theory, this research underscores the necessity of a supportive athletic ecosystem that prioritizes the athlete’s psychological health. It is essential to acknowledge that the scope of these conclusions is specific to youth individual sports within the Chinese context and should be interpreted as one significant component of a multifaceted etiology of athletic distress. Ultimately, the results suggest that mitigating competitive anxiety requires not only the moderation of controlling external demands but also the active nourishment of the athlete’s sense of mastery and volitional freedom.

## Data Availability

The raw data supporting the conclusions of this article will be made available by the authors, without undue reservation.
